# Subscapularis CT-Scan Evaluation in Patients with Proximal Humerus Fracture: Reverse Total Shoulder Arthroplasty Versus Hemi-Arthroplasty

**DOI:** 10.3390/jcm14155257

**Published:** 2025-07-24

**Authors:** Edoardo Gaj, Andrea Redler, Alessandro Maggiori, Susanna Pagnotta, Natale Criseo, Vikranth Mirle, Matthew Daggett, Angelo De Carli

**Affiliations:** 1 Rome Jewish Hospital, 00186 Rome, Italy; edoardogaj@gmail.com; 2 Orthopedic Department, S. Andrea Hospital, University of Rome “Sapienza”, 00185 Rome, Italy; ale.magg.93@gmail.com (A.M.); susannapagnotta@gmail.com (S.P.); natalecriseo@libero.it (N.C.); angelo.decarli@gmail.com (A.D.C.); 3 Midwest Orthopaedics at Rush, Rush University Medical Center, Chicago, IL 60612, USA; vikranthmirle@uchicagomedicine.org; 4 Sano Orthopaedics, Kansas City University, MO 64106, USA; matthewdagget@gmail.com

**Keywords:** CT, HA, humeral fracture, RTSA, subscapularis tendon

## Abstract

**Background/Objectives**: Hemiarthroplasty (HA) and Reverse Total Shoulder Arthroplasty (RTSA) are both reliable treatment options for complex proximal humerus fractures. The role of the subscapularis tendon is well-defined in HA, whereas it plays a controversial role in RTSA. The purpose of our study is to evaluate its role in patients with proximal humerus fractures treated with HA and RTSA and investigate its association with clinical outcomes. **Methods**: Sixty-eight consecutive patients with proximal humeral fracture were prospectively enrolled into the study from June 2015 to May 2020 (RTSA = 36; HA = 32). Pre- and postoperative shoulder CT scans were performed to measure the subscapularis muscle cross-sectional area (SMCSA) and the supraspinatus fossa cross-sectional area (SFCSA). The SMCSA/SFCSA ratio was employed to normalize measurements against individual patient anatomy. Patient reported outcomes (PROs) and range of motion (ROM) were evaluated at the final follow-up. **Results**: The RTSA group demonstrated superior patient-reported outcomes (PROs) and range of motion (ROM) compared to the HA group. Notably, the Constant Score was significantly higher in the RTSA group (58.00 vs. 38.50; *p* = 0.0001), as well as forward flexion (147.50° vs. 90.00°; *p* < 0.0001). A postoperative reduction in subscapularis size of >35% occurred more frequently in RTSA patients (55.6%) than in HA patients (25%) (*p* = 0.01). The loss of subscapularis surface was greater in the RTSA patients (*p* = 0.018). **Conclusions**: RTSA demonstrated better results compared to HA, providing better ROM and PROs. Postoperative reduction in subscapularis size was significantly higher in RTSA compared to HA. Subscapularis condition seems to show no correlation with functional outcome in RTSA.

## 1. Introduction

Proximal humeral fractures represent the third most common type of osteoporotic fracture [1,2]. Hemiarthroplasty (HA) and Reverse Total Shoulder Arthroplasty (RTSA) are both reliable treatment option for complex proximal humerus fractures; the former is largely influenced by tuberosities healing [3], whereas the latter is less dependent on rotator cuff to achieve good functional results [4]. Specifically, RTSA emerges as a viable therapeutic option for 3- or 4-part complex proximal humeral fractures in patients over 65, contributing to satisfactory functional recovery [5].

While subscapular tendon repair is essential for shoulder stability and function in HA [6,7,8], its role in RTSA is debated. Some studies suggest a role of the subscapularis repair in improving the stability of the implant and range of motion (ROM) [9,10]; whereas others associate subscapularis repair with impaired glenohumeral external rotation (ER) and increased demand on deltoid muscle during arm elevation [11,12].

Several previous studies have examined the importance of subscapular repair in both HA and RTSA. De Carli et al. [13] demonstrated that in patients treated with RTSA and subscapularis repair for eccentric osteoarthritis, the size of subscapularis muscle was significantly reduced in the majority of patients. To our knowledge, no previous studies have examined the importance of subscapularis condition on functional outcomes comparing hemiarthroplasty and reverse shoulder replacement in patients with complex proximal humerus fractures.

The primary aim of this study, which is exploratory and hypothesis-generating, is to evaluate subscapularis muscle condition in both HA and RTSA with pre- and post-operative CT scans. The secondary aim is to analyze the clinical outcome of both prosthesis types and compare the results with the post-operative subscapularis condition.

## 2. Materials and Methods

### 2.1. Study Population

This study underwent University Ethics Committee approval (protocol code n. 67/2007; date of approval 10 December 2007). Between June 2015 and May 2020, one hundred and seventy-eight consecutive patients accessed the emergency department of our hospital with proximal humeral fractures. Fifteen patients declined their consent to participate. One hundred and sixty-three patients provided their informed consent to participate and were enrolled into this prospective non-randomized study.

Inclusion criteria:

1. Age greater than 60;
2. Proximal humeral fracture with displaced three- or four-part (according to Neer’s classification [3]) confirmed by shoulder X-ray and pre-operative CT scan;
3. Absence of neurological disease or cognitive dysfunction.

Exclusion criteria:

1. Fracture-dislocation;
2. History of rheumatic diseases;
3. Presence of neurological disease or cognitive dysfunction;
4. Pathologic or open fractures;
5. Associated neurovascular injury;
6. Degenerated or irreparable subscapularis tendon.

Pre-operative rotator cuff tendons were classified according to Goutallier and Zanetti using CT [14,15]. RTSA was the treatment of choice in patients with compromised rotator cuff tendons (high degree of atrophy (Zanetti 3) and adipose infiltration (Goutallier 3-4), comminution of the great tuberosity or in patients with limited ROM and symptomatic nocturnal shoulder pain prior to the fracture.

### 2.2. Surgical Technique

RTSA and HA were performed using the SMR reverse shoulder system (LIMA Corporate, Udine, Italy) with patients in beach chair positions. The senior authors performed all the operations. No cement was used.

In RTSA, the deltopectoral approach was performed in all cases to have a better view of the baseplate inclination and to minimize the risk of axillary nerve injury. Tenotomy of the subscapularis was performed at the level of the anatomical neck. The tendon was considered reparable if it could be reattached to its original insertion with transosseous suture with mild force at 20° of ER and neutral abduction. All RTSA procedures used a 40-mm or 44-mm glenosphere with the humerus at 10° of retroversion. Prostheses were secured by press-fit fixation. The greater tuberosity was repaired in every patient.

In HA, the deltopectoral approach was performed in all cases. Subscapularis and supraspinatus were secured with sutures to provide temporary fixation of the tuberosities. If lesser tuberosity was not fractured, an osteotomy was performed. Tenotomy of the long head of the biceps tendon was performed, and the glenoid fossa was inspected to rule out any injury after retrieving the humeral head. Infraspinatus tendon was secured with sutures. The prosthesis was positioned and secured by press-fit fixation after measuring the humeral head. The tuberosities were fixed by non-absorbable suture through the bone and the corresponding holes in the prosthesis.

### 2.3. Clinical Evaluation

Patients of both groups were evaluated at the final follow-up (minimum of two years) by a standardized examiner uninvolved in the arthroplasty procedure.

Active shoulder range of motion (ROM) was evaluated with external (ER) and internal (IR) rotation, with the arm in abduction, adduction, and forward flexion.

Posterior IR was recorded on a 6-point vertebral segment related to scale [16]: (1) dorsum of hand positioned at lateral thigh; (2) buttock; (3) lumbosacral junction; (4) waist (3rd lumbar vertebra); (5) 12th dorsal vertebra; (6) interscapular region.

All patients were required to complete Constant score, Quick Dash, Simple Shoulder Test, and Visual Analog Scale (VAS) pain questionnaires at the end of the follow-up.

### 2.4. CT Evaluation

A shoulder CT scan was performed at the time of the diagnosis and the end of the follow-up in both groups by the same radiologist.

**Figure 1:**
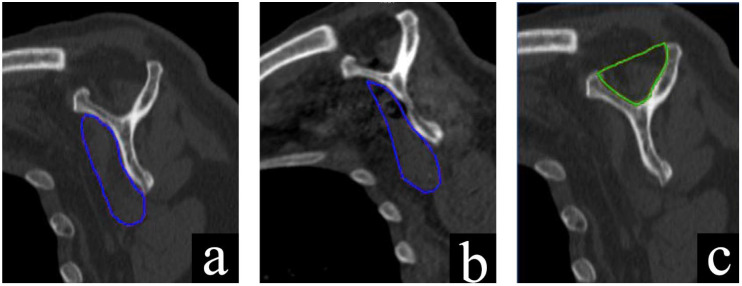
(**a**) Pre-operative delineation of SMCSA; (**b**) Post-operative delineation of SMCSA; (**c**) Delineation of SFCSA.

Toshiba Aquilion 16-slice CT scanner (Tokyo, Japan) was used to obtain images for all patients with the same protocol, calibration method, and scanning settings (120 kVp, 125 mAs, field of view of 250 mm, and a detector pitch of 15). A 512 matrix of 1-mm-thick slices was created using a soft-tissue filter and raster artifact suppression tool (slice overlap: 0.5 mm). The typical Y-shaped image [17] was created by reconstructing the sagittal-oblique CT slice from the original CT data sets. The evaluation was conducted at the most lateral portion, where the scapular spine was still in continuity with the scapular body. The quantitative analysis of the muscle cross-sectional areas was performed using Awserver 3.4 GE Healthcare, Milwaukee, WI, USA. The measurements were obtained through a manual segmentation technique. To ensure consistency and reduce inter-observer variability, all delineations were performed by the same experienced radiologist, who was blinded to the surgical group and clinical outcomes of the patients. For each measurement, the radiologist manually traced the boundaries of the subscapularis muscle belly (for SMCSA) and the supraspinatus fossa (for SFCSA) on the selected sagittal-oblique slice (the “Y-view”). The muscle boundaries were defined by visually identifying the fascial planes separating the muscle from adjacent adipose tissue and bone structures. The segmentation was based entirely on anatomical landmarks to ensure the accuracy of the delineation. The software then automatically calculated the area within the traced contour in square millimeters. The cross-sectional areas of the subscapularis muscle (SMCSA) and supraspinatus fossa (SFCSA) were measured in square millimeters. The SMCSA/SFCSA ratio was employed to account for individual anatomical differences between patients [18] (Figure 1).

Each group (HA and RTSA) was classified into three groups according to their SMCSA/SFCSA ratio: <1.5, 1.5–2, or >2.0. Postoperative reduction in subscapularis size was evaluated at the final follow-up according to the SMCSA/SFCSA ratio groups, the absolute value of subscapularis cross-sectional area (mm^2^), and the absolute SMCSA reduction (i.e., <25%, 25–35% and >35% reduction) compared to their pre-operative values.

### 2.5. Statistical Analysis

Statistical analysis was performed by an independent statistician. Parametric variables were expressed as medians. Frequencies and percentages were used to assess the distribution of nonparametric variables. Wilcoxon-Mann-Whitney test was used to analyze differences in ROM, clinical scores, and absolute reduction in SMCSA between the two groups. The chi-square test and Fisher’s exact test were used to analyze differences in non-parametric variables. Statistical analysis was performed using IBM SPSS Statistics 24 (Armonk, NY, USA) and JMP Pro 12 (Cary, NC, USA). Statistical significance was defined as *p* < 0.05. Additionally, a post hoc power analysis was performed to assess the adequacy of the sample size and the robustness of key statistical comparisons. This analysis was conducted after data collection, based on observed group sizes and effect sizes, with a significance threshold set at α = 0.05.

## 3. Results

### 3.1. Demographics

Of the 163 patients initially enrolled in the study, 49 met at least one exclusion criterion and were subsequently excluded. Of the 49 excluded patients, nine had a fracture-dislocation of the humeral head, 14 reported a history of rheumatic disease, 18 suffered neurological disease or cognitive dysfunction, five had a pathological fracture, and three had a neurovascular injury. An additional 11 patients were treated conservatively due to a high risk of anesthesiologic complications. Of the 103 remaining patients, 52 were scheduled to undergo RTSA and 51 HA based on pre-operative assessment indications. 15 patients were excluded after surgery due to an intra-operative finding of severely degenerated subscapularis tendon, which made it impossible to suture (seven in the RTSA group and eight in HA group). Additionally, 20 patients were lost to clinical or radiological follow-up (nine in the RTSA group and 11 in the HA group), leaving a total of 36 patients who underwent RTSA (aged 71.3 ± 4.4, range 66–88 years), and 32 underwent HA (aged 69.5 ± 5.1, range 64–81 years). At the time of surgery, the mean age among all patients was 70.9 ± 6.3 years (range 64–88 years) Fracture classification for each group is shown in Table 1.

In the HA-group all patients were right-hand dominant (100%) while in the RTSA-group 34 were right-hand-dominant (94.44%) and two were left-hand-dominant (5.56%). The operative shoulder in the HA-group was the right in 14 subjects (43.75%) and the left in 18 (56.25%), in the RTSA-group was the right in 20 subjects (55.56%) and the left in 16 (44.44%). No statistically significant difference was found between the two groups concerning the dominant limb (*p* = 0.4943) or the operative shoulder (*p* = 0.3311).

The two groups did not show any statistically significant difference in the pre-existing risk factors: hypertension was found in 22 (61.11%) patients in the RTSA group vs. 20 (62.50%) in the HA group (*p* = 0.90), diabetes mellitus in four (11.11%) patients in the RTSA group vs. six (18.75%) in the HA group (*p* = 0.49), and smoking habits in four (11.11%) patients in the RTSA group vs. two (6.25%) in the HA group (*p* = 0.67).

Assessment of preoperative shoulder function was precluded, as it was infeasible to evaluate in patients presenting with existing fractures.

### 3.2. Clinical Assessment

Patients in RTSA group reported better results in all PROs except for the VAS score which was slightly higher in the HA group. Statistically significant differences in Constant Score, Quick Dash, simple shoulder test and VAS score were found (Table 2).

### 3.3. Range of Motion

RTSA patients achieved greater degrees of ER and IR (both in adduction and abduction), forward flexion, and abduction and demonstrated statistically significant better results on the 6-point vertebral segments scale compared to the HA patients (*p* = 0.0046) (Table 3).

### 3.4. Radiographic Results

**Figure 2:**
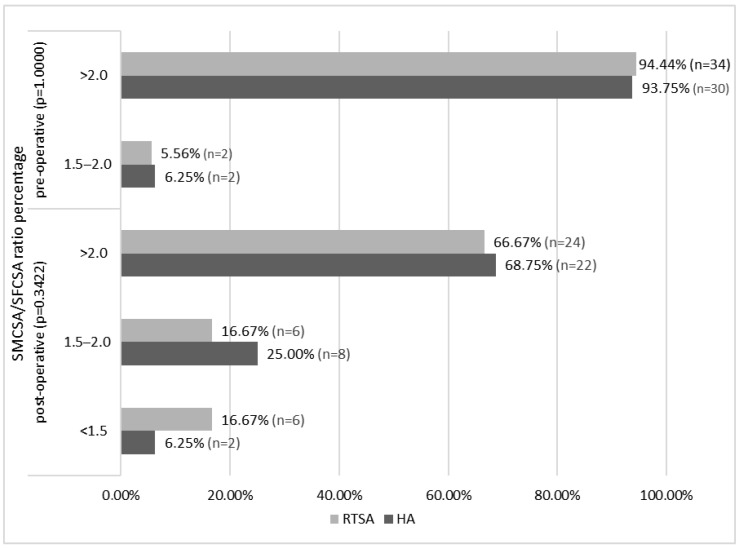
Pre and post-operative SMCSA/SFCSA ratio comparison in HA and RTSA patients.

Pre- and post-operative assessment comparisons of the SMCSA/SFCSA ratio in both groups are shown in Figure 2; no statistically significant differences were found between the two groups.

The pre-operative median surface area of subscapularis was greater in absolute value in the RTSA patient group (RTSA 1643 ± 638.22 mm^2^ vs. HA 1294 ± 683.21 mm^2^) (*p* = 0.031); however, the difference between the pre- and post-operative subscapularis surface was greater in the patients who underwent RTSA (RTSA −682.5 ± 561.32 mm^2^ vs. HA −338.5 ± 416.25 mm^2^) (*p* = 0.0186), indicating a larger loss in the subscapularis surface following surgery.

**Figure 3:**
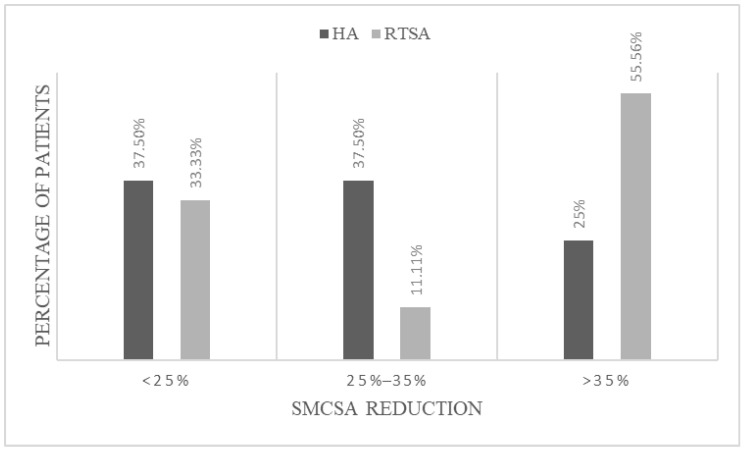
Comparison of post-operative SMCSA reduction percentage in HA and RTSA (*p* = 0.0115).

All patients presented with a post-operative SMCSA reduction (*p* = 0.0115). Specifically, within the HA group, 37.5% (12) of patients showed a SMCSA reduction of <25%, 37.5% (12) of patients experienced a reduction between 25% and 35%, and 25% (8) of patients had a greater than 35% reduction. On the other hand, within the RTSA cohort, 33.3% (12) of patients had a SMCSA reduction of <25%, 11.1% (4) of patients experienced a reduction of 25–35%, and 55.6% (20) of patients had >35% reduction (Figure 3). Based on the observed proportions and the respective group sizes, the statistical power was calculated at 74.3% (α = 0.05). Although slightly below the conventional 80% threshold, this result indicates a moderate-to-high confidence in the reliability of the observed difference.

## 4. Discussion

The main finding of the study was the increased reduction in the subscapularis cross-sectional area after surgery identified in the RTSA cohort when compared to the HA cohort. Patients treated with RTSA had a larger median surface area of the subscapularis pre-operatively; whereas, after surgery, the subscapularis area decreased more than in the HA group. Additionally, more RTSA patients showed reductions >35% in SMCSA than HA patients. Furthermore, our study demonstrated significantly better results in the RTSA group with respect to patient-reported outcome metrics (Constant Score, Quick Dash, and Simple Shoulder Test results) and physical exam findings (IR, ER, forward flexion, and abduction) when compared to the HA group. Notably, the postoperative VAS scores, while low overall, were significantly higher in the RTSA group compared to the HA group. This result could be a chance finding or a statistical anomaly, given the limited sample size of our study. Future research should aim to clarify this observation.

The implementation of HA and RTSA as a treatment option for proximal comminuted humeral fractures has been extensively studied in prior literature. A meta-analysis from Wang et al. [19] identified that patients who underwent RTSA had a lower rate of complications, better tuberosity healing, larger active forward elevation, and higher ASES score when compared with HA patients. Similarly, Cuff DJ et al. [20] demonstrated that positive final clinical outcomes and ROM values for elderly patients treated with HA for acute comminuted proximal humeral fractures depended on greater tuberosity healing, which was not always likely in this patient population due to poor bone quality. Conversely, positive outcomes in the RTSA-treated patients were less contingent upon the patient’s bone quality, and thus these patients had more consistent and superior results. However, it is important to note that the literature also reports excellent outcomes with hemiarthroplasty in carefully selected patients with good bone quality and repairable tuberosities [21].

Although the subscapularis had a greater loss in size, the clinical exam findings (ROM) and clinical survey scores were significantly better in the RTSA group. This potentially supports the hypothesis that the post-operative condition of the subscapularis does not affect the clinical outcome in patients treated with RTSA. De Boer et al. [22] performed an ultrasound examination of the subscapularis tendon following an RTSA surgery and discovered an intact subscapularis tendon in only 40% of the patients who had the subscapularis repaired with no differences in ROM and strength between these two patient groups.

The increased reduction in subscapularis surface area in RTSA compared to HA noted in this study could be explained by changes in the biomechanics of the shoulder. In the normal shoulder, the subscapularis rotates the humerus differently depending on whether it is above or below the center of rotation [23]. Following RTSA, the biomechanics of the shoulder are changed; the humerus moves inferiorly, and consequently, the subscapularis tendon is shifted inferiorly to the center of rotation, changing the muscle traction vector [24]. Subsequently, the subscapularis functions as an adductor for the first 70° of the arm abduction in post-RTSA patients [25]. Furthermore, medially translating the humerus shortens the subscapularis muscle and limits its ability to generate active movement [26,27,28].

The subscapularis reduction can be secondary to normal rotator cuff degeneration and fatty infiltration, as this is common in an elderly population [29]. However, the finding of a major loss in subscapularis size following RTSA, when compared to HA, may support our previous biomechanical hypothesis or may be a consequence of the surgical procedure.

Prior literature delineates other advantages of subscapularis repair, with several studies reporting a reduced dislocation rate for non-lateralized RTSA [9,30]. According to Routman [23], lateralization of the prosthesis may have a different impact on how the subscapularis repair affects stability or function. An increase in IR ROM [31] was also reported as a result of the subscapularis repair; however, in a lateralized design this may cause excessive tension on the subscapularis, leading to a consequent loss in ER ROM.

Strengths of this study first include its prospective design, along with the paucity of literature analyzing the importance of the subscapular size on CT scans in the postoperative results for RTSA and HA. Second, all surgical procedures were performed by the same expert surgeon with a standardized implant and surgical technique. Lastly, despite a lack of randomization, the RTSA and HA groups did not show any significant difference with respect to limb dominance, operative laterality, the pre-operative SMCSA/SFCSA ratio, and the presence of comorbidities.

This study also has some limitations. The main limitation of this study is the non-randomized design; the surgical allocation criteria, which assigned patients with more compromised rotator cuffs to the RTSA group, may have led to an associated bias in the selection of patients. Secondly, the exclusion of 15 patients intraoperatively due to an irreparable subscapularis tendon introduced a post-hoc selection bias, likely removing the most severe cases from both cohorts and potentially artificially improving the reported functional outcomes. An additional methodological weakness is the lack of an inter- and intra-rater reliability analysis for the CT measurements, which reduces their objectivity. Furthermore, the sample size is relatively small, with 68 total patients. It must be noted that pre-operative clinical scores were not obtained because all patients were admitted to the hospital with an acute shoulder injury. Finally, the high mean age of the patients is associated with an increased frequency of proximal humeral fractures in that age range; thus, the results might not be applicable to younger patients.

## 5. Conclusions

In conclusion, postoperative subscapular size was found to be significantly reduced in patients treated with RTSA compared to patients treated with HA. However, better functional results in terms of ROM and a higher degree of patient satisfaction were reported for RTSA patients despite the subscapularis condition, although the SMCSA/SFCSA ratio didn’t show any significant differences between the two groups.

Our study suggests that, while in HA subscapularis repair appears mandatory to achieve good results, as shown by the smaller reduction in subscapularis size after surgery, in RTSA, this may not be correlated with better ROM due to the biomechanical changes that occur with this type of prosthesis. Further studies should be conducted to confirm the controversial role of subscapularis in RTSA.

## Figures and Tables

**Table 1:** Demographic of fracture types according to Neer’s and AO Classification.

	Neer 3	Neer 4	AO 11-B1	AO 11-B2	AO 11-C1	AO 11-C2
HA group (*n* = 32)	18 (56.25%)	14 (43.75%)	10 (31.25%)	8 (25.00%)	5 (15.62%)	9 (28.12%)
RTSA group (*n* = 36)	21 (58.3%)	15 (41.7%)	11 (30.55%)	10 (27.78%)	7 (19.44%)	8 (22.22%)
Tot.	39	29	21	18	12	17

**Table 2:** Comparison between median post-operative clinical scores (±SD) outcomes for patients undergoing HA and RTSA.

Variable	HA	RTSA	*p* Value
Constant Score	38.50 ± 11.73	58.00 ± 16.23	0.0001
Quick Dash	27.25 ± 23.10	14.75 ± 9.34	0.0006
Simple Shoulder Test	4.50 ± 14.75	8.00 ± 15.80	0.0129
VAS (0–10)	0.00 ± 2.12	2.00 ± 2.04	0.0311

**Table 3:** Comparison of post-operative ROM degrees (±SD) between patients undergoing HA and RTSA.

		HA	RTSA	*p* Value
Forward flexion		90.00 ± 37.64	147.50 ± 39.74	<0.0001
Abduction		85.00 ± 37.87	95.00 ± 37.83	0.0175
ER	Add	25.00 ± 13.26	45.00 ± 22.93	0.0011
	Abd	30.00 ± 13.18	30.00 ± 26.12	0.0258
IR	Add	35.00 ± 10.55	42.50 ± 19.51	0.0047
	Abd	30.00 ± 13.56	42.50 ± 24.38	0.0257
Posterior IR	Lateral side of the thigh	0 (0%)	2 (5.56%)	0.0046
	Buttock	16 (50%)	6 (16.67%)	
	Lumbosacral junction	10 (31.25%)	8 (22.22%)	
	Waist	2 (6.25%)	12 (33.33%)	
	Lumbothoracic junction	4 (12.5%)	8 (22.22%)	

## Data Availability

The data presented in this study are available on request from the corresponding author due to privacy, legal or ethical reasons.

## Abbreviations

The following abbreviations are used in this manuscript:

HA	Hemiarthroplasty
RTSA	Reverse Total Shoulder Arthroplasty
SMCSA	Subscapularis muscle cross-sectional area
SFCSA	Supraspinatus fossa cross-sectional area
PROs	Patient reported outcomes
ROM	Range of motion
CT	Computed Tomography
ER	External rotation
IR	Internal rotation
VAS	Visual Analog Scale
ASES	American Shoulder and Elbow Surgeons score
